# Ionic liquid-based ultrasonic-assisted extraction coupled with HPLC to analyze isoquercitrin, trifolin and afzelin in *Amygdalus persica* L. flowers

**DOI:** 10.1186/s13065-023-01018-w

**Published:** 2023-08-19

**Authors:** Wei Zhang, Zhenhua Yin, Qingfeng Guo, Lin Chen, Juanjuan Zhang

**Affiliations:** 1 Comprehensive Utilization of Edible and Medicinal Plant Resources Engineering Technology Research Center, Huanghe Science and Technology College, Zhengzhou, 450063 Henan Province China; 2 Zhengzhou Key Laboratory of Synthetic Biology of Natural Products, Huanghe Science and Technology College, Zhengzhou, 450063 China; 3 Henan Joint International Research Laboratory of Drug Discovery of Small Molecules, Huanghe Science and Technology College, Zhengzhou, 450063 China

**Keywords:** *Amygdalus persica* L., Ionic liquid, HPLC, Response surface methodology, Isoquercitrin

## Abstract

This study aimed to establish a method for the simultaneous determination of isoquercitrin, trifolin and afzelin in *A. persica* flowers by high performance liquid chromatography (HPLC) with ionic liquid as extractant and ultrasonic-assisted extraction. The effects of ionic liquid concentration, solid–liquid ratio, number of crushing mesh, ultrasonic time, extraction temperature, and ultrasonic power on the extraction yield of three target compounds were investigated using the extraction yield of target analytes as the index. According to the results of single factor experiment, the Box-Behnken design-response surface methodology (BBD) was used to optimize the extraction method and compared with the traditional extraction method. The results showed that, calibration curves had excellent linearity (*R*^2^ > 0.9990) within the test ranges. In combination with other validation data, this method demonstrated good reliability and sensitivity, and can be conveniently used for the quantification of isoquercitrin, trifolin and afzelinin *A. persica* flowers. And the contents of isoquercitrin, trifolin and afzelin were 64.08, 20.55 and 75.63 μg/g, respectively. The optimal process obtained by BBD was as follows: ionic liquid concentration was 1.0 mol/L, solid–liquid ratio was 1:40 g/ml, mesh sieve was 50 mesh, ultrasonic time was 40 min, extraction temperature was 50 °C, and ultrasonic power was 400 W. Under the optimal conditions, the theoretical predicted total extraction yield of the three target compounds was 159.77 μg/g, which was close to the actual extraction value (160.26 μg/g, *n* = 3), this result indicating that the optimal process parameters obtained by response surface methodology analysis were accurate and reliable. The method was simple, accurate and rapid for determination the contents of three active ingredients in *A. persica* flowers.

## Introduction

**Fig. 1:**
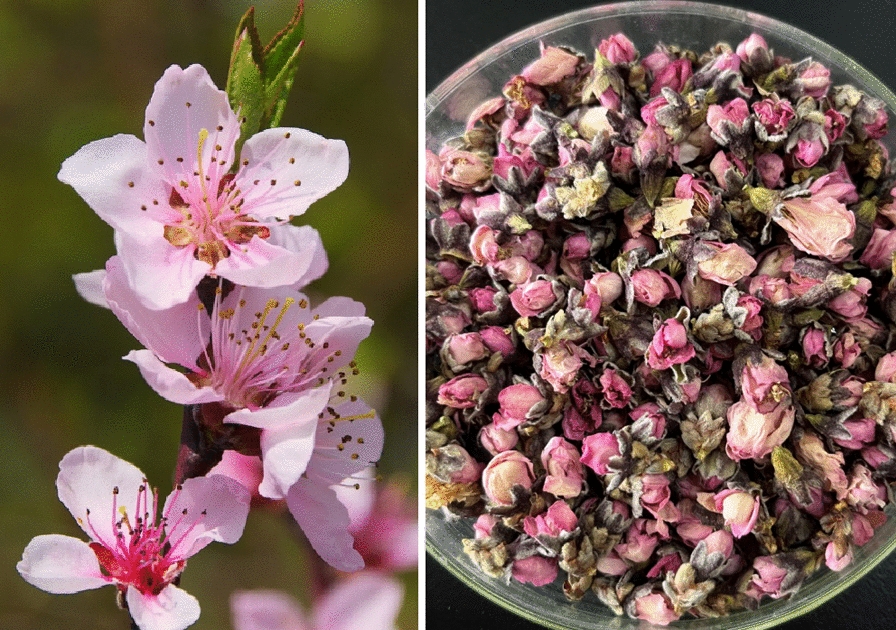
*Amygdalus persica* flowers

*Amygdalus persica* L., belonging to Rosa ceae, is widely distributed in most regions of China, and with the effect of reducing diarrhea and defecation, promoting water and reducing swelling (Fig. 1) [1]. *A. persica* flowers contain flavonoids, polyphenols, polysaccharides, carotenoids, vitamins, amino acids and trace elements [2, 3], which has beauty and health care functions, and is widely used in the field of food and medicine. The development of *A. persica* flowers wine [4], essential oil [5], cake [6, 7], and other products have been reported. In addition, *A. persica* flowers are currently consumed as a tea for weight loss in China. The studies show that *A. persica* flowers exerts anti-obesity effects in obese mice and these beneficial effects might be mediated through improved hepatic lipid metabolism by reducing lipogenesis and increasing fatty acid oxidation [8, 9]. Previous studies have revealed several pharmacological effects of *A. persica* flowers, including protection of the skin from ultraviolet radiation [10, 11], inhibition of melanogenesis [12], stimulation of intestinal motility [13], antioxidant and antibacterial activities [14, 15].

As an environment-friendly solvent, compared with water and conventional organic solvents, ionic liquids had the characteristics of high ionic conductivity, low volatility, strong polarity, good dissolving and extracting ability, and high chemical stability. Ionic liquids could dissolve cellulose and destroy plant cell walls, and had a broad application prospect in the field of extraction and separation of natural products [16–18]. Ultrasonic-assisted extraction had been proved to significantly shorten the extraction time and improve the extraction yield in the process of assisting plant extraction. In the process of ultrasonic application, cavitation and mechanical effects would be produced, which made it easier for solvents to penetrate into plant raw materials, destroy plant cell walls and promote the release of active components [19–21]. Ionic liquid-based ultrasonic-assisted extraction was a green extraction technology with high efficiency and energy saving, which combines the characteristics of high efficiency cavitation effect generated by ultrasound and high solubility of ionic liquid [22]. The technology had been applied to the extraction and isolation of *Paris polyphylla* saponins [23], flavonoids [24], acacetin [25], pectin [26] and other active components. Box-Behnken design-response surface methodology (BBD-RSM) was a widely used method to optimize experimental conditions in recent years [27]. And BBD-RSM was a two-order experimental design method based on three levels. It adopted multivariate quadratic equation to fit the functional relationship between factors and response values, and seeked the optimal process parameters by analyzing the regression equation. In the process of optimization, software was used to optimize the whole experimental conditions with respect to the fitting degree, SNR and the significance factors of the equation, so as to seek the best extraction process parameters [28, 29].

However, there is still no related report about the ionic liquid-based ultrasonic-assisted extraction of compositions in *A. persica* flowers. In our previous study, the chemical constituents and coagulation activity of *A. persica* were investigated. The results of coagulation activity showed that isoquercitrin, trifolin and afzelin could remarkably shorten activated partial thromboplastin time, prothrombin time, thrombin time and reduce the content of fibrinogen, and had procoagulant activity in vitro [30]. Thus, on this basis, this study aimed to establish a method for the simultaneous determination of isoquercitrin, trifolin and afzelin in *A. persica* flowers by high performance liquid chromatography (HPLC) with ionic liquid as extractant and ultrasonic-assisted extraction.

## Materials and reagents

### Instruments

All the analyses were performed on a Waters 2695 liquid chromatography system (Waters, Milford, USA) equipped with a vacuum degasser, a quaternary solvent deliver system, an autosampler, a column compartment, and a w2489 UV visible detector. Kq-250db CNC ultrasonic cleaner was purchased from Kunshan Ultrasonic Instrument Co., LTD (Kunshan, China). AG285 electronic analytical balance was purchased from Mettler Toledo (Switzerland). FZ102 plant sample mill (Huanghua Zhongxing Instrument Co., LTD., Hebei). KQ-500DB ultrasonic cleaner (Kunshan Ultrasonic Instrument Co., LTD., Jiangsu). Design-Expert software (Version 8.0.6, Stat-Ease. Inc., Minneapolis, MN. USA).

### Chemicals and reagents

1-butyl-3-methylimi-dazolium bromide ([BMIM]Br), 1-butyl-3-methylimidazolium tetrafluorob-orate ([BMIM]BF4), 1-butyl-3-methylimidazolium hexafluorophosphate, ([BMIM]PF6), 1-hexyl-3-methylimidazolium hexafluorophosphate ([HMIM]PF6) were purchased from Tokyo Chemical industry Co., Ltd., Tokyo, Japan.

Isoquercitrin, trifolinand and afzelin were provided by Henan Engineering Research Center for Comprehensive Utilization of Edible and Medicinal Plant Resources, Huanghe Science and Technology College, and their purities were up to 98%. Deionized water was prepared using a Milli-Q ultrapure water purifier (ELGA, Labwater, Marlow, UK). Acetonitrile and methyl alcohol were purchased from Thermo Fisher Technologies LTD. All other reagents were analytical grade.

### Plant material

*A. persica* flowers samples were obtained from the Pharmacy of Zhang Zhongjing, in June 2022, and identified by Professor Changqin Li of Henan University. The voucher specimens were deposited in the Institute of Natural Medicine of Huanghe Science and Technology College.

## Methods

### Preparation of solutions

#### Standard solutions

The Standard solutions were prepared according to the reference [31], and the concentrations of isoquercitrin, trifolin, and afzelin were 211.3, 192.7 and 236.6 μg/mL, respectively. The concentrations of isoquercitrin, trifolin, and afzelin of the mixed standard solution were 84.4, 38.5 and 94.4 μg/mL, respectively.

#### Sample solutions

The sample solutions of *A. persica* flowers were prepared according to the reference [31]. *A. persica* flowers were extracted by ultrasonic extraction with a certain amount of methanol after drying and crushing. And methanol was used as blank control solution.

### Chromatographic conditions

Waters e2695 HPLC system (Waters, Milford, USA) was used for the chromatographic analysis. The analytical column was an XTERRA MS C18 column (4.6 mm × 250 mm, 5 µm) (Waters, Milford, USA). The mobile phase was 0.1% formic acid solution (A)—acetonitrile (B) with gradient elution (0–10 min, 10–25% B; 10–37 min, 10–25% B; 37–41 min, 35–100% B; 41–45 min, 100–10% B). The flow rate was 1.0 mL/min and column temperature was maintained at 25 °C. The detection wavelength was set at 360 nm. The injection volume was 10 μL with needle wash.

### System suitability

**Fig. 2:**
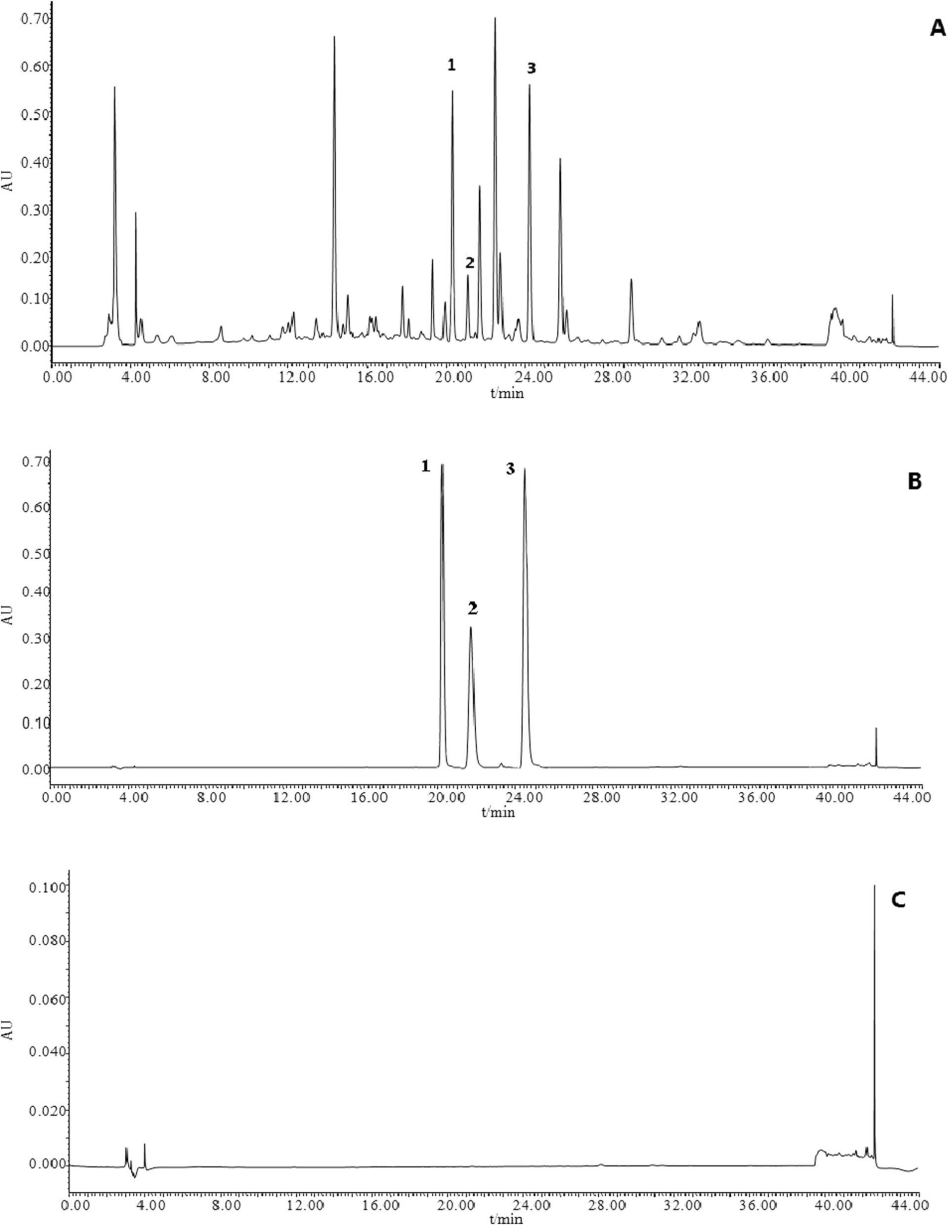
HPLC chromatograms of *A. persica* flowers (**A**), reference substances (**B**) and methanol blank control (**C**). *Note* 1. isoquercitrin; 2. trifolin; 3. afzelin

According to the reference [31], standard solutions, sample solution and methanol blank control solution were taken for sample injection and determination to analyze system suitability according to chromatographic conditions. All results were obtained in acceptable ranges (Fig. 2).

### Investigation of linear relations

According to the reference [31], regression equation and each coefficient (*R*^2^) were obtained using standard solutions. The results were presented in Table 1. All the marker compounds showed good linearity within the test range (*R*^2^ > 0.9990).

**Table 1:** Regression equation and linear range of three active ingredients

Components	Regression equation	*R*^2^	Linear range (μg/mL)
Isoquercitrin	y = 1785328x − 169635	0.9998	0.0537–0.6122
Trifolin	y = 3560729x − 341706	0.9996	0.0902–0.8461
Afzelin	y = 652743x − 55403	0.9995	0.04500–1.1935

### Optimization of ultrasonic assisted extraction technology

After selecting the extractant, ionic liquid concentration (0.2, 0.4, 0.6, 0.8 and 1.0 mol/L), solid–liquid ratio (1:20, 1:30, 1:40, 1:50 and 1:60 g/mL), mesh number (20, 30, 40, 50 and 60 mesh), ultrasonic time (20, 30, 40, 50 and 60 min), extraction temperature (30, 40, 50, 60 and 70 °C) and ultrasonic power (100, 200, 300, 400 and 500 W) were used as single factor experimental conditions to investigate the effect of single factor on the extraction efficiency of three compounds in *A. persica* flowers. And optimize each single factor. On this basis, combined with Box–Behnken design-response surface experiment, the extraction technology was optimized.

## Results

### Selection of dispersant

**Fig. 3:**
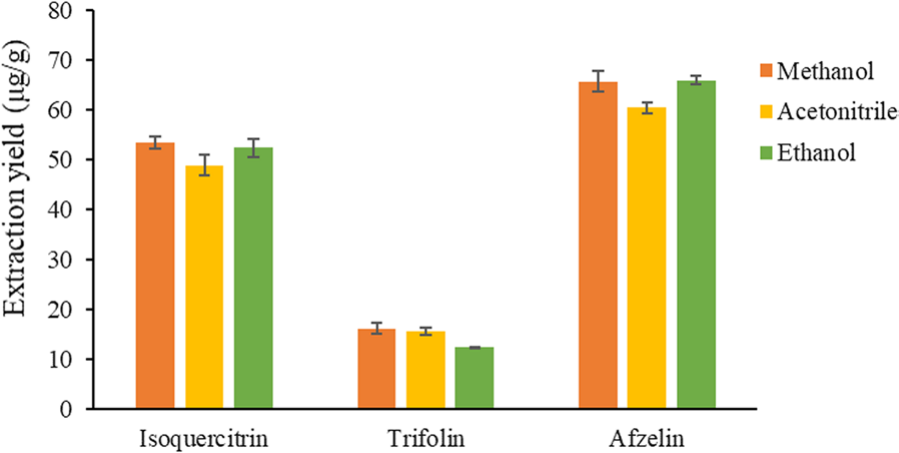
Influence of dispersant on the extraction yields of three target analytes

In this test, methanol, acetonitrile and ethanol were selected for extraction, and the results showed that methanol had the highest extraction yield of target compounds (Fig. 3). Then, on this basis, ionic liquid was added to further improve the extraction yield of the target compounds in the sample.

### Selection of ionic liquid

**Fig. 4:**
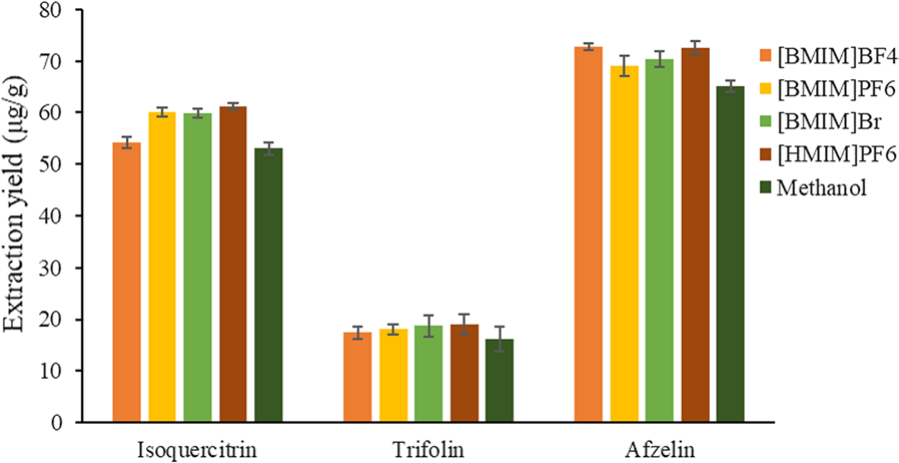
Effect of the type of ionic liquid on the extraction yields of three target analytes

The difference of extraction solution had great influence on the extraction yield of active components. In this test, 0.6 mol/L [BMIM]BF4, [BMIM]PF6, [BMIM]Br, [HMIM]PF6 methanol solutions and pure methanol solution were selected as the extraction solution. After crushing, the *A. persica* flowers were screened 40 mesh, the solid–liquid ratio was 1:30 g/mL, the ultrasonic time was 40 min, the extraction temperature was 30 °C and the ultrasonic power was 200 W. The extraction effect of different ionic liquids on the target analytes were compared, and the results were shown in Fig. 4.

The results showed that the extraction yield of the four ionic liquid solutions for the target analytes were significantly higher than that of methanol solution, and the extraction yield of [HMIM]PF6 methanol solution for isoquercitrin and trifolin were the highest, and the extraction yield of [BMIM]BF4 and [HMIM]PF6 for afzelin were the similar. Therefore, [HMIM]PF6 was selected as the extraction solvent for the follow-up study.

### Analysis of single factor experimental

#### Selection of ionic liquid concentrations

On the basis of the above optimization factors, other conditions being the same, using 0.2, 0.4, 0.6, 0.8 and 1.0 mol/L [HMIM]PF6 methanol solution as the extraction agent, the effect of ionic liquid concentration on the extraction yields of the target analytes were compared.

**Fig. 5:**
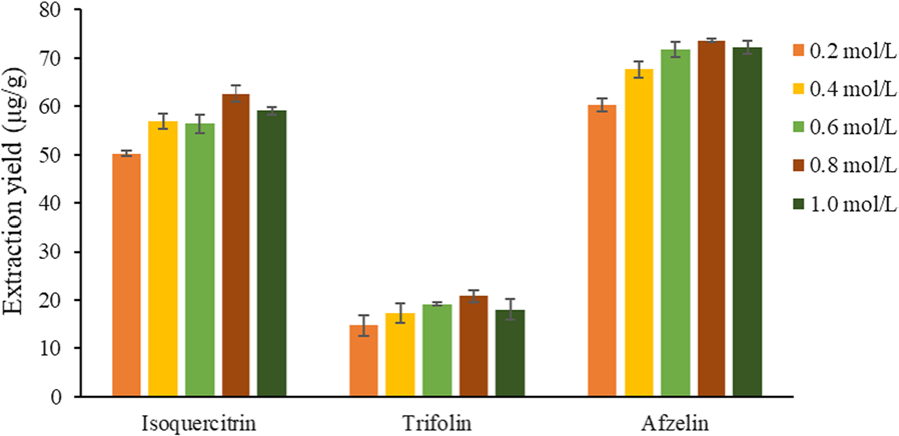
Effect of ionic liquid concentrations on the extraction yields of three target analytes

In Fig. 5, when the ionic liquid concentration was 0.8 mol/L, the extraction yield of the target analytes reached the maximum value. However, as the ionic liquid concentration continued to increase, the extraction yields gradually decreased. The reason may be related to the viscosity of the extraction solution. If the concentration of ionic liquid was too high, the viscosity of the extraction solution will also increase, and the diffusion ability of the extractant will become worse, which makes it difficult for the extractant to penetrate into the interior of *A. persica* flowers cells, thus reducing the dissolution ability of [HMIM]PF6 to the target analytes [32]. Therefore, the concentration of [HMIM]PF6 was selected as 0.8 mol/L.

#### Selection of solid–liquid ratio

Based on the above optimization conditions, the effects of solid–liquid ratios of 1:60, 1:50, 1:40, 1:30 and 1:20 g/mL on the extraction yield of the target analytes were investigated.

**Fig. 6:**
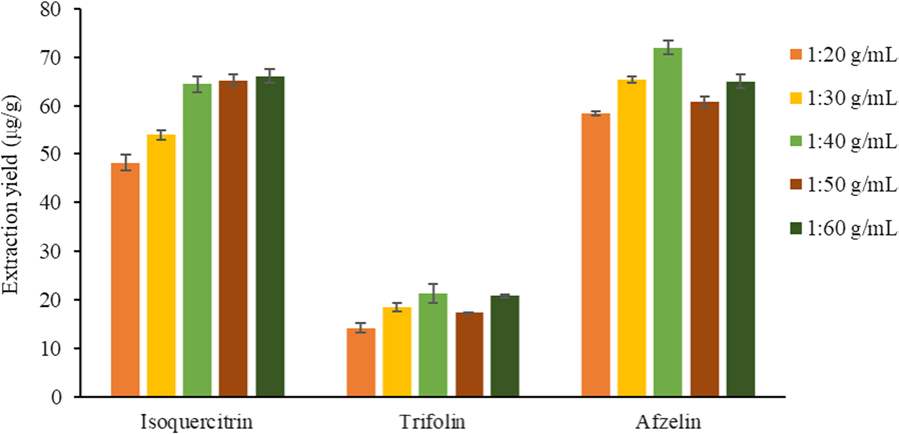
Effect of solid–liquid ratio on the extraction yield of three target analytes

As shown in Fig. 6, with the increase of solid–liquid ratio, the extraction yields of the three compounds gradually increased. When the solid–liquid ratio was 1:40 g/mL, the extraction yield of isoquercitrin increased slowly. However, the extraction yield of trifolin and afzelin reached the maximum, and the extraction yields tended to decrease as the solid–liquid ratio continued to increase. It may be that as the solid–liquid ratio continues to increase, the osmotic pressure of the solvent increases, resulting in the dissolution of other impurities and a decrease in the extraction yields of the target analytes. Too small solid–liquid ratio will lead to incomplete extraction of active components in the medicinal materials, while too large will lead to waste of solvents. Therefore, the solid–liquid ratio of 1:40 g/mL was selected.

#### Selection of mesh sieve

**Fig. 7:**
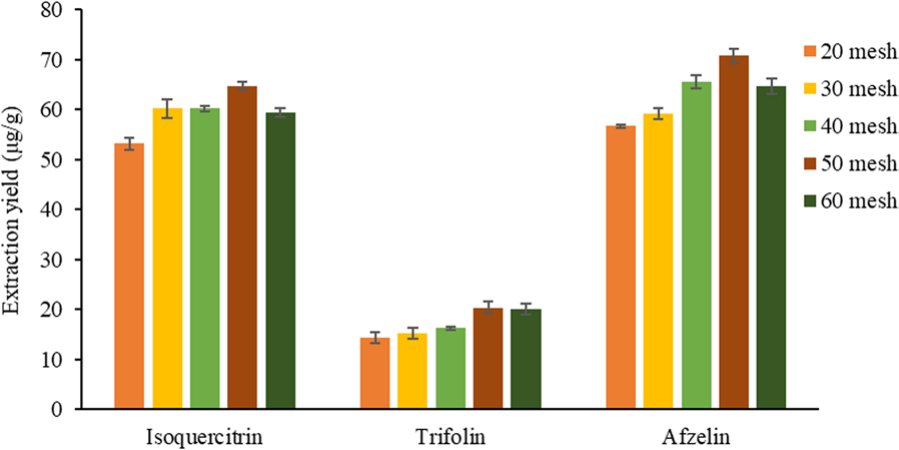
Effect of mesh sieve on the extraction yields of three target analytes

During the extraction of the effective components of traditional Chinese medicine, the size of the medicinal material had a great influence on the extraction yield. The smaller the particle size, the larger the effective contact area with the solvent and the higher the extraction yield. On the basis of the above optimization conditions, the influence on the extraction yield of the target analytes were investigated when the number of minced mesh were 20, 30, 40, 50 and 60 mesh, respectively. The results were shown in Fig. 7.

In Fig. 7, the 50 mesh sampling sieve had the highest extraction yield for the target analyte, because the smaller the powder particle size, the easier it is to extract the active ingredients contained in the sample. However, when the powder particle size was too small, it was easy to be condensed into clusters by ionic liquid, thus preventing the release of the active components in the samples. Therefore, the sifted mesh number was 50.

#### Selection of ultrasonic time

Based on the above optimization conditions, the influence of extraction time of 20, 30, 40, 50 and 60 min on the yield of target analytes were investigated.

**Fig. 8:**
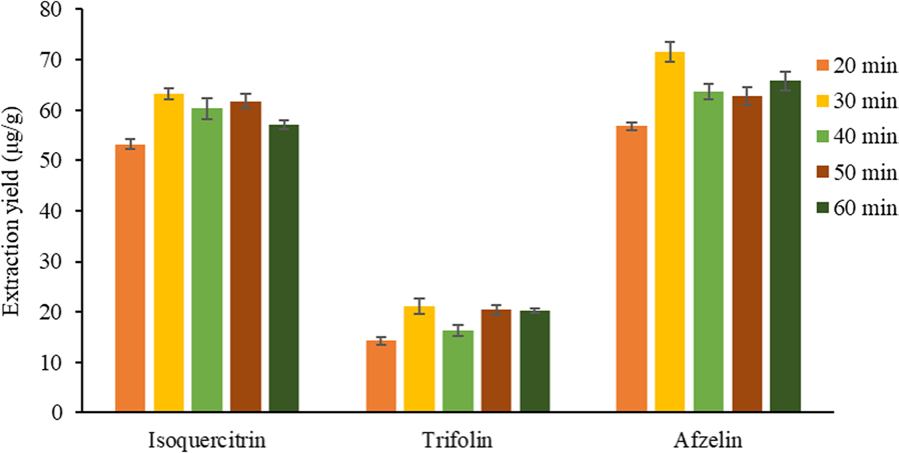
Effect of ultrasonic time on the extraction yield of three target analytes

In Fig. 8, with the increase of ultrasonic extraction time, the extraction yield of the target extracts increased. When the ultrasonic extraction time increased to 30 min, the extraction yield reached the peak. The ultrasonic extraction time continued to increase, and the extraction rate tended to be gentle and decreased. The reason may be that the target extracts basically reached saturation and no longer dissolve obviously. In addition, too long extraction time will destroy the structure of flavones, and the extraction yield will decrease [33]. Therefore, 30 min of ultrasonic extraction was selected as the optimal condition in this experiment.

#### Selection of extraction temperature

**Fig. 9:**
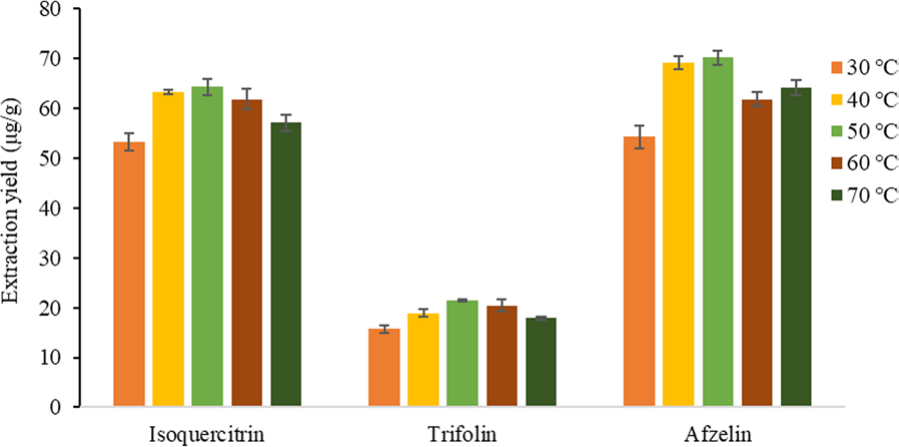
Effect of extraction temperature on the extraction yield of three target analytes

Based on the above optimization conditions, the influence of extraction temperature at 30, 40, 50, 60 and 70 °C on the extraction yield of the target analytes were investigated. The result was shown in Fig. 9.

With the increase of extraction temperature, the extraction yield of target analytes gradually increased. When the extraction temperature was 50 °C, the extraction yield reached the maximum, but the temperature continued to rise, and the extraction rate showed a trend of decreasing. The reason may be that with the increase of the temperature of the system, the movement rate and frequency of the ionic liquid are increasing, which makes it easier to penetrate into the peach blossom tissue cells, accelerate the dissolution of the target analytes, and thus increase the extraction rate of the target analyte [34]. However, if the temperature of the system continues to increase, the structure of the target analyte would be destroyed. It was also possible that with the increase of temperature, the dissolution of other contents in the cell increases, resulting in the increase of solution viscosity, which affects the outward dissolution of the target analyte, thus reducing the extraction yield. Therefore, the extraction temperature of 50 °C was selected.

#### Selection of ultrasonic power

Based on the above optimization conditions, the influence of ultrasonic power of 100, 200, 300, 400 and 500 W on the extraction yield of the target analytes were investigated.

**Fig. 10:**
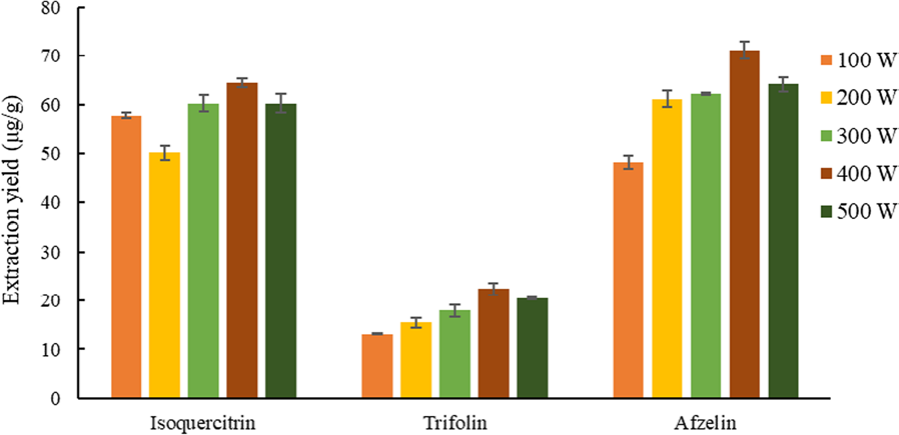
Effect of ultrasonic power on the extraction yield of three target analytes

The results were shown in Fig. 10. When the ultrasonic power was 400 W, the extraction rate of the three target compounds reached the maximum. With the increase of ultrasonic power, the breaking effect of ultrasonic on plant cells gradually increased, which could accelerate the dissolution of target components. However, when the destructive power exceeds a certain limit, the dissolved impurities also increase, and the extraction rate does not increase significantly but shows a downward trend. Therefore, ultrasonic power of 400 W was selected.

### Box–Behnken design-response surface experiment

Synthesize the above single factor experiments, ionic liquid concentration (A), solid–liquid ratio (B), number of crushing mesh (C) and extraction time (D) were selected as independent variables of the response surface model. The fixed ultrasonic temperature and ultrasonic power were 50 °C and 400 W, respectively. The total extraction yield of three compounds from *A. persica* flowers was taken as the evaluation index and designed by Design-Expert 8.0.6 Trail software. Each factor involved three levels of high, low and medium, which were represented by 1, 0 and − 1, respectively. The variable levels were shown in Table 2 and the test results were shown in Table 3.

**Table 2:** BBD for the independent variables and corresponding response values

Level	Independent variable			
	A (mol/L)	B (g/mL)	C (mesh)	D (min)
− 1	0.6	30	40	20
0	0.8	40	50	30
1	1.0	50	60	40

**Table 3:** Experimental design and results of BBD

Run order	A (mol/L)	B (g/mL)	C (mesh)	D (min)	Total extraction yield (mg/g)
1	0	0	0	0	157.31
2	0	0	1	1	159.62
3	0	0	0	0	157.15
4	− 1	0	− 1	0	156.34
5	1	0	− 1	0	157.63
6	0	1	− 1	0	156.14
7	0	0	0	0	158.64
8	0	1	0	1	158.53
9	0	− 1	0	1	157.21
10	1	0	0	− 1	158.83
11	0	0	− 1	1	158.24
12	0	1	1	0	156.12
13	0	− 1	1	0	157.3
14	0	0	0	0	157.83
15	− 1	0	0	1	158.32
16	− 1	1	0	0	157.12
17	0	0	0	0	158.41
18	1	1	0	0	158.04
19	0	1	0	− 1	156.82
20	− 1	− 1	0	0	155.72
21	0	− 1	− 1	0	154.64
22	1	0	0	1	159.37
23	0	0	1	− 1	156.93
24	− 1	0	1	0	157.65
25	1	0	1	0	158.71
26	− 1	0	0	− 1	156.12
27	0	0	− 1	− 1	156.42
28	0	− 1	0	− 1.000	156.54
29	1	− 1	0	0.000	157.59

Design-Expert 8.0.6 Trail software was used to carry out quadratic polynomial stepwise regression fitting on the test data, and the quadratic multiple regression equation was established: *Y* = 106.83 + 9.53A + 1.22B + 0.83C − 0.18D − 0.12AB − 0.03AC − 0.21AD − 6.70BC + 2.60BD + 2.18CD + 0.4.12A^2^ − 0.01B^2^ − 5.48C^2^ + 3.54D^2^. The variance analysis of regression model is shown in Table 4. *P* < 0.0001 of the regression model indicated that the regression model was extremely significant. The missing fitting item *P* > 0.05 was not significant, indicated that the model was valid. The coefficient of determination *R*^2^ of the model was 0.9027, and the coefficient of determination* R*^2^_Adj_ = 0.9054. The coefficient of determination *R*^2^ of the model and the coefficient of determination *R*^2^_Adj_ of correction were both high and close to each other, indicated that the model had high accuracy and universality, and could be used to analyze and predict the effect of ionic liquid on the extraction of three target compounds.

**Table 4:** ANOVA for the fitted quadratic polynomial model for optimization of extraction parameters

Source	Sum of squares	df	Mean square	*F* value	*P* value
Model	33.91	14	2.42	9.27	< 0.0001
A	6.60	1	6.60	25.28	0.0002
B	1.18	1	1.18	4.54	0.0514
C	3.99	1	3.99	15.28	0.0016
D	7.73	1	7.73	29.59	< 0.0001
AB	0.23	1	0.23	0.86	0.3684
AC	0.013	1	0.013	0.051	0.8252
AD	0.69	1	0.69	2.64	0.1266
BC	1.80	1	1.80	6.88	0.0201
BD	0.27	1	0.27	1.04	0.3262
CD	0.19	1	0.19	0.72	0.4090
A^2^	0.18	1	0.18	0.67	0.4254
B^2^	7.07	1	7.07	27.07	0.0001
C^2^	1.95	1	1.95	7.45	0.0163
D^2^	0.81	1	0.81	3.10	0.0999
Residual	3.66	14	0.26		
Lack of fit	1.94	10	0.19	0.45	0.8598
Pure error	1.72	4	0.43		
Cor total	37.57	28			

Highly significant (*P* < 0.01); Significant (*P* < 0.05)

The primary terms A, C, D and B^2^ of the model had extremely significant effects on the total extraction yield of the three target compounds (*P* < 0.01), and the BC had significant effects on the total extraction yield (*P* < 0.05). There was no significant difference between primary term B, AB, AC, AD, BD, CD, A^2^, C^2^ and D^2^ (*P* > 0.05). The *F* value of single factor also reflected the influence degree of each factor on the total extraction yield of target compounds. According to the results, the factor that had the greatest influence on the total extraction yield was the solid–liquid ratio (D).

**Fig. 11:**
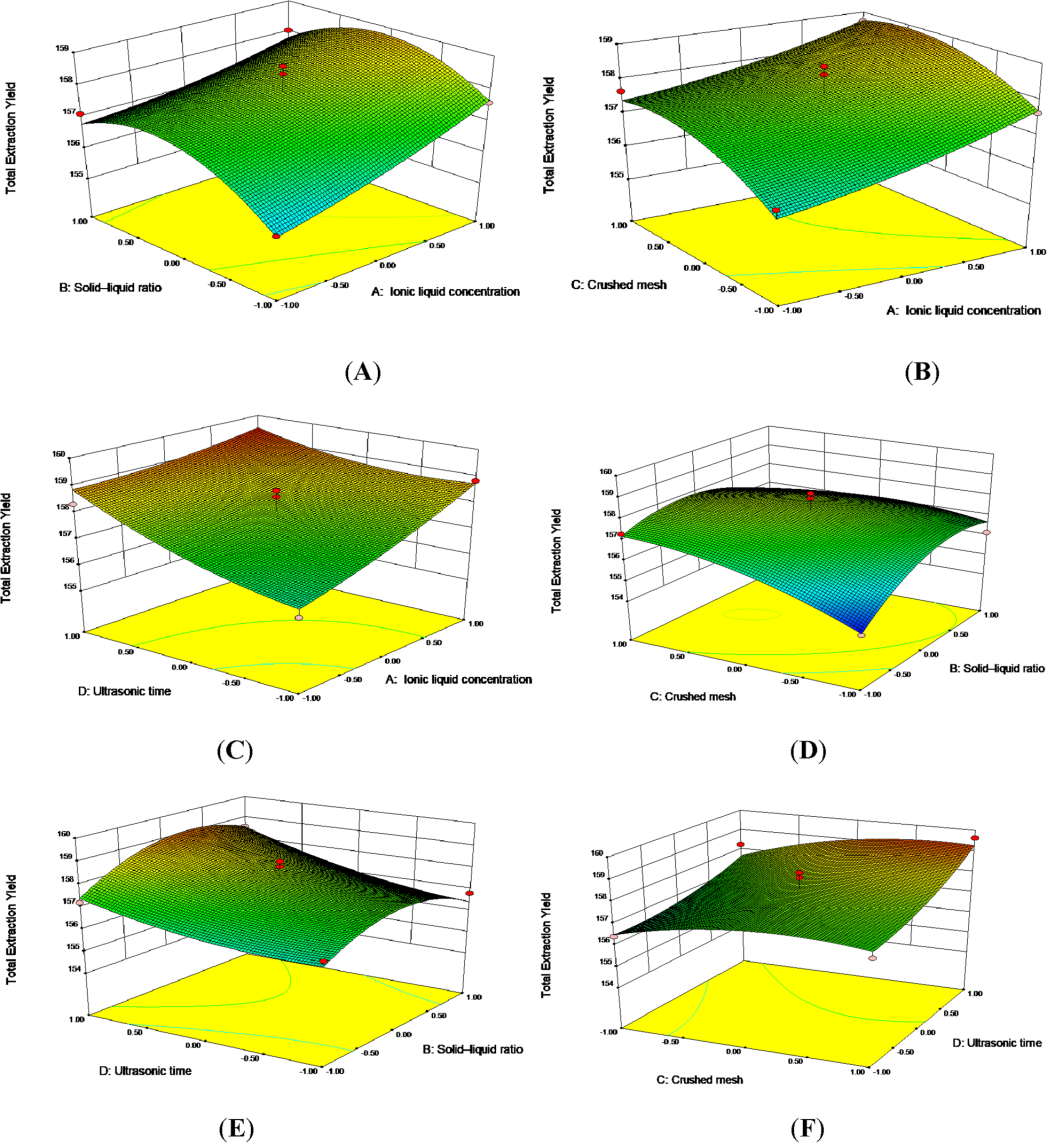
The interactive effect of solid-liquid ratio and ionic liquid concentration (**A**), number of crushing mesh and ionic liquid concentration (**B**), extraction time and ionic liquid concentration (**C**), number of crushing mesh and solid-liquid ratio (**D**), extraction time and solid-liquid ratio (**E**), number of crushing mesh and extraction time (**F**) on the total extraction yield of three compounds from *A. persica* flowers

The response surface diagram could significantly reflect the strength of interaction between various factors. The Design-Expert 8.0.6 Trail software was used to analyze the response surface diagram, and the influence of the interaction of various factors on the response value can be obtained. The results were showed in Fig. 11. If the response surface was steeper, it indicated that the factor had a greater influence on the total extraction yield of the target analytes, and the response value was more sensitive to the change of extraction conditions. If the response surface was relatively flat, it indicated that the fluctuation of this factor had little influence on the response value. In Fig. 11, it could be seen from the steepness of the response surface diagram that the interaction of BC factor was the strongest, while that of AD factor was the weakest. With the increase of solid–liquid ratio, the total extraction rate of the three compounds increased slowly to the maximum value and then decreased gradually. The total extraction rate increased with the increase of ionic liquid concentration. The extraction yield increased with the increase of the number of crushing mesh, and then the extraction rate changed little with the increase of the number of crushing mesh.

The regression equation was further analyzed by the Design-Expert 8.0.6 Trail software, and the optimal process parameters were obtained as follows: ionic liquid concentration was 1.0 mol/L, solid–liquid ratio was 1:40 g/ml, number of crushing mesh was 50 mesh, ultrasonic time was 40 min, extraction temperature was 50 °C, and ultrasonic power was 400 W. Under the optimal conditions, the theoretical predicted total extraction yield of the three target compounds was 159.77 μg/g, which was close to the actual extraction value (160.26 μg/g, *n* = 3), this result indicating that the optimal process parameters obtained by response surface methodology analysis were accurate and reliable.

### Method validation

Precision test, repeatability test, stability test and recovery test indicated that the the three active ingredients (isoquercitrin, trifolin and afzelin) in *A. persica* flowers achieved better separation. They had good linearity in a certain mass concentration range (*R*^2^ > 0.9990), good precision (RSD < 1.79%), repeatability (RSD < 1.80%) and stability (RSD < 2.32%), and the average recoveries (n = 6) were 98.72–99.25% with RSD of 0.83–2.41%. The established HPLC method was simple to operate, high sensitivity, stable and reliable, and had good repeatability. It can be used for quality control and evaluation of *A. persica* flowers. The contents of isoquercitrin, trifolin and afzelin in *A. persica* flowers were 64.08, 20.55 and 75.63 μg/g, respectively.

## Discussion

According to the reference [35, 36], the three components were scanned in the wavelength range of 200–400 nm, and the three components had a better linear relationship at 360 nm. Meanwhile, the separation effect of various mobile phase systems was investigated, and the influence of different mobile phase systems on the chromatographic peak separation degree, peak time and peak shape was analyzed. Finally, acetonitrile-0.1% formic acid solution was selected as the mobile phase.

In this study, methanol solution with ionic liquid added had a higher extraction yield for the target analytes than pure methanol solution, because ionic liquid, as a new solvent, had a higher solubility for cellulose, and the primary cell wall of Chinese medicinal plants was mainly composed of cellulose, which was the main obstacle to solvent extraction of bioactive components. The dissolution of cellulose promoted the dissolution of active ingredients and improved the extraction efficiency [37]. In addition, ionic liquid had high viscosity and low mass transfer efficiency during extraction of pure ionic liquid. Therefore, ionic liquid aqueous solution and methanol solution were commonly used as extractant. In this way, the viscosity of extractant was reduced, mass transfer was enhanced, energy consumption was reduced, and extraction rate was improved.

By comparing the effects of [BMIM]BF4, [BMIM]PF6, [BMIM]Br, [HMIM]PF6 methanol solution on extraction yields of isoquercitrin, trifolin and afzelin, it was found that [HMIM]PF6 had more advantages than the other three ionic liquids. Wu et al. compared the extraction effect of five ionic liquids with different chain lengths on tanshinone, tanshinone I and tanshinone IIA under ultrasonic assistance, and found that the short-chain ionic liquid was ineffective for tanshinone extraction, and the extraction effect was effective only when the chain length was longer than 10, and the longer the alkyl chain of ionic liquid, the stronger the extraction ability of tanshinone [38]. Wei et al. investigated the extraction rate of saponins from quinoa by a series of imidazole ionic liquids with different compositions, and found that the extraction rate was the highest when the carbon chain length of the ionic liquid was 4 [39]. Ma et al. showed that the extraction rate of alkaloids increased with the increase of cationic alkyl side chain length of ionic liquid, and ionic liquid with chain-like side chain had better extraction ability of alkaloids [40]. It could be seen that both the cation and anion of ionic liquid have influence on the extraction efficiency. However, the specificity and regularity of different types of ionic liquids for the extraction of different types of active ingredients have not been found, which needs further research.

## Conclusion

In this study, the contents of isoquercitrin, trifolin and afzelin in *A. persica* flowers were determined by high performance liquid chromatography (HPLC) with ionic liquid as extractant and ultrasonic-assisted extraction technology for the first time and the optimal extraction conditions were determined by single factor test and response surface method. And under the optimal technological conditions, the contents of isoquercitrin, trifolin and afzelin in *A. persica* flowers were 64.08, 20.55 and 75.63 μg/g, respectively. Environmentally friendly reagent was used as extraction agent, which improved extraction efficiency, avoided the pollution of organic solvent to the environment, and reduced the harm to human body. And it had important reference significance for the innovation of extraction methods of active ingredients of traditional Chinese medicine. However, the research on the mechanism of extraction and separation of ionic liquid and its action law are not clear at present. Therefore, in the future research work, the interaction between *A. persica* flowers and the molecular structure of ionic liquid will be further studied based on the molecular model, and the action law of ionic liquid extraction of compounds will be further clarified.

## Data Availability

The datasets used and/or analyzed during the current study are available from the corresponding author on reasonable request.
